# Changes in Antimicrobial Resistance in Pediatric Urinary Pathogens Before, During, and After the COVID-19 Pandemic

**DOI:** 10.3390/antibiotics14121243

**Published:** 2025-12-09

**Authors:** Seon Hee Lim, Kyo Jin Jo, Shin Yun Byun, Yun-Jin Lee, Su Eun Park, Ji Yeon Song

**Affiliations:** Department of Pediatrics, Pusan National University Yangsan Hospital, Pusan National University School of Medicine, Yangsan 50612, Republic of Korea; kkrsh@naver.com (S.H.L.); godjkj@nate.com (K.J.J.); byun410@hanmail.net (S.Y.B.); jinnyeye@naver.com (Y.-J.L.); psepse@naver.com (S.E.P.)

**Keywords:** pediatric UTI, antimicrobial resistance, ciprofloxacin, extended-spectrum β-lactamase, COVID-19, interrupted time-series

## Abstract

Background: Pediatric urinary tract infections (UTIs) are increasingly complicated to treat due to antimicrobial resistance (AMR). The coronavirus disease 2019 (COVID-19) pandemic has led to substantially reduced pediatric antibiotic prescribing, but its impact on resistance trends remains unclear. This study aimed to investigate the AMR trends in urinary pathogens from children under 24 months of age hospitalized with febrile UTI during the pre-, during-, and post-COVID-19 pandemic periods. Methods: We conducted a retrospective study of children aged <24 months who were hospitalized at a tertiary center in Korea between 2008 and 2023 for first febrile UTI. The patients were stratified by age (<100 days vs. 100 days to 24 months) and pandemic period (pre-, during-, and post-COVID-19). Interrupted time-series (ITS) analysis and multivariable logistic regression were used to assess the temporal trends and predictors of ciprofloxacin nonsusceptibility. Results: Ciprofloxacin susceptibility decreased significantly during the pandemic, especially among infants < 100 days. ITS analysis demonstrated an immediate 12.1% increase in ciprofloxacin nonsusceptibility at pandemic onset, which persisted thereafter. Extended-spectrum β-lactamase production was the strongest predictor of ciprofloxacin resistance. Conclusions: These findings suggest that adult antibiotic use and clonal dissemination may contribute to the persistence and spread of AMR, highlighting the need for integrated stewardship and genomic surveillance.

## 1. Introduction

Urinary tract infection (UTI) is one of the most common serious bacterial infections in children, affecting approximately 7% of febrile infants and often leading to hospitalization in the first 2 years of life [1]. The increasing burden of antimicrobial resistance (AMR) has complicated the management of pediatric UTI [2,3]. The coronavirus disease 2019 (COVID-19) pandemic has substantially altered antibiotic use. Although bacterial co-infections are uncommon, most hospitalized COVID-19 patients received antibiotics, raising concerns about inappropriate broad-spectrum use [4,5]. In contrast, multiple studies have documented substantial reductions in pediatric antibiotic use during the pandemic. In Korea, outpatient pediatric antibiotic prescriptions declined by 52% in 2020 compared with 2019 [6], and national surveillance data showed a 17.7% reduction in overall antibiotic use in 2020 compared with that in 2015, while pediatric respiratory antibiotic prescriptions declined by 14–18% [7]. This decline likely reflects the impact of social-distancing measures, school closures, and universal masking, which markedly reduced respiratory and gastrointestinal viral infections and, consequently, the need for empirical antibiotics [8,9]. However, this decline in pediatric antibiotic use occurred within the context of Korea’s already high AMR burden. Korea represents a high-AMR setting compared with other OECD countries. National surveillance data have reported third-generation cephalosporin and fluoroquinolone resistance in *Escherichia coli* (*E. coli*) exceeding 35–40% [10] and ampicillin and trimethoprim/sulfamethoxazole (TMP/SMX) resistance in pediatric urinary isolates over 60% [11]. These rates are substantially higher than those reported in most OECD countries, where ampicillin and TMP/SMX resistance in pediatric urinary *E. coli* isolates typically remain below 50% [2]. Such findings highlight Korea’s high baseline AMR burden and raise an importance of continued surveillance in pediatric populations. Because pediatric and adult urinary pathogens largely overlap, pediatric AMR trends may be shaped not only by pediatric prescribing but also by broader community-level antimicrobial pressures and cross-age transmission dynamics [12,13]. Therefore, we investigated temporal trends in AMR among urinary pathogens isolated from children under 24 months of age hospitalized with febrile UTI during the pre-, during-, and post-COVID-19 pandemic periods, to clarify whether observed changes reflect broader ecological influences beyond pediatric prescribing.

## 2. Results

### 2.1. Baseline Characteristics

Between January 2008 and 31 August 2023, 1149 patients were included, comprising 849 (73.9%) males and 300 (26.1%) females. The mean age at onset was 121.1 ± 91 days, with 605 patients (52.7%) diagnosed before 100 days of age and 544 patients (47.3%) diagnosed between 100 days and 24 months of age. The mean duration of fever was 2.3 ± 1.4 days. Kidney ultrasonography showed anomalies in 64% of patients, while dimercaptosuccinic acid scans detected cortical defects in 37.7% of patients (Supplementary Table S1). When divided into early-onset and delayed-onset groups, the proportion of males was higher in the early-onset group than in the delayed-onset group (85% vs. 61.6%, *p* < 0.001). Fever duration was longer in the delayed-onset group (2.9 ± 1.6 days), and inflammatory markers, including WBC, ESR, and CRP, were significantly higher in the delayed-onset group (*p* < 0.01, Table 1). Imaging studies revealed that urinary structural anomalies were more frequent in the early-onset group than in the delayed-onset group (67.4% vs. 59.9%, *p* = 0.027), whereas cortical defects were more prevalent in the delayed-onset group (31.0% vs. 43.9%, *p* < 0.001, Table 1).

### 2.2. Antibiotic Susceptibility by Age Group

Urinary pathogens included *Citrobacter* spp., *E. coli*, *Enterobacter cloacae*, *Enterococcus* spp., *Klebsiella* spp., *Morganella morganii*, and *Proteus mirabilis*, with *E. coli* accounting for 89.6% of cases. No difference in pathogen distribution was observed according to the age of onset (Supplementary Tables S2 and S3). Ciprofloxacin susceptibility was significantly higher in the early-onset group. For most antibiotics, except for gentamicin and TMP/SMX, susceptibility was higher in the early-onset group, but the differences were not statistically significant (Table 2).

### 2.3. Antibiotic Susceptibility by COVID-19 Period

**Figure 1:**
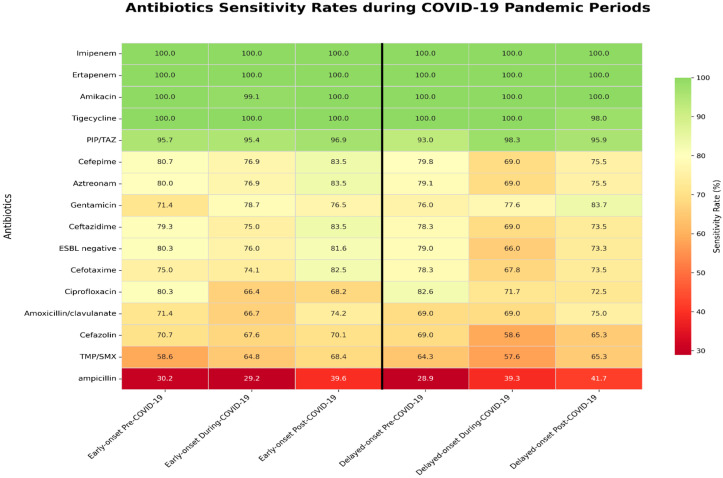
Differential antibiotic sensitivity between early-onset and delayed-onset groups during the COVID-19 pandemic. Coronavirus disease 2019; TMP/SMX, trimethoprim and sulfamethoxazole; PIP/TAZ, piperacillin and tazobactam; ESBL, Extended-spectrum β-lactamase.

When comparing antibiotic susceptibility across the pre-, during-, and post-COVID-19 periods, amoxicillin/clavulanate susceptibility significantly decreased during the COVID-19 period (*p* = 0.021). Aztreonam and ciprofloxacin, which are less commonly used in pediatric patients, also showed a significant decrease in susceptibility (*p* < 0.001 and *p* = 0.003, respectively). Ciprofloxacin susceptibility decreased during the COVID-19 period, but modestly increased in the post-COVID-19 period (Table 3). In the early-onset group, ciprofloxacin susceptibility declined from 80.3% in the pre-COVID-19 period to 66.4% during the pandemic, with only partial recovery to 68.2% post-COVID-19 (*p* = 0.026). In contrast, the delayed-onset group showed no significant changes in antibiotic susceptibility (Figure 1).

### 2.4. Interrupted Time-Series Analysis (ITS)

**Figure 2:**
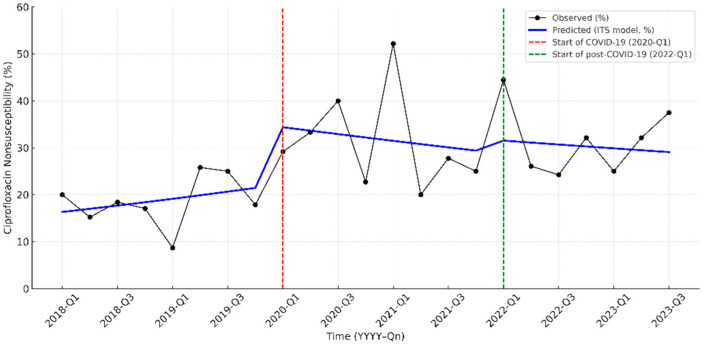
Interrupted time-series analysis of ciprofloxacin nonsusceptibility in pediatric UTI pathogens from 2018 to 2023. The solid black line indicates the observed nonsusceptibility, while the blue line represents the predicted trend by the interrupted time-series (ITS) model. The red dashed line marks the start of the COVID-19 pandemic period (2020-Q1), and the green dashed line indicates the beginning of the post-COVID-19 period (2022-Q1).

Before the pandemic, ciprofloxacin nonsusceptibility showed a modest but not significant upward trend. At the onset of the COVID-19 pandemic (2020-Q1), there was an immediate increase of 12.1 percentage points (95% CI, 2.8–23.7; *p* = 0.019). During the pandemic period, no significant slope change was detected (β = −0.081 per quarter; 95% CI, −0.187 to 0.025; *p* = 0.135), indicating stabilization after the initial surge. At the second breakpoint (2022-Q1), no additional level or slope changes were observed (all *p* > 0.05) (Figure 2; Supplementary Table S4).

### 2.5. Multivariable Analysis

**Figure 3:**
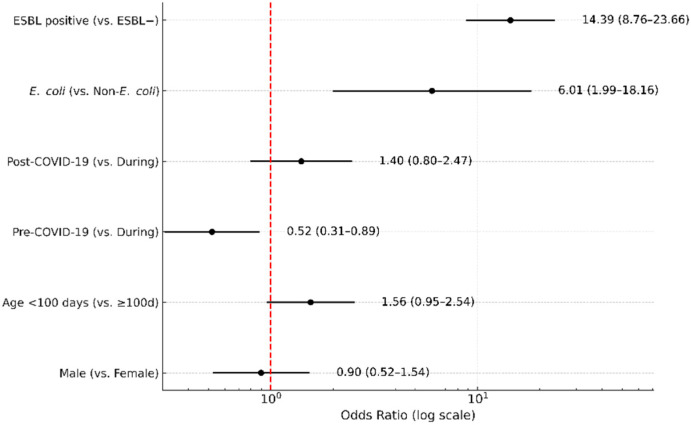
Multivariable logistic regression for predictors of ciprofloxacin nonsusceptibility. Adjusted odds ratios (ORs) with 95% confidence intervals (CIs) are shown for extended-spectrum β-lactamase (ESBL) production, bacterial species (*E. coli* vs. non-*E. coli*), COVID-19 period (pre vs. during, post vs. during), age (<100 days vs. ≥100 days), and sex (male vs. female). The red dashed vertical line represents the null value (OR = 1).

In the multivariable logistic regression, extended-spectrum β-lactamase (ESBL) production was the strongest independent predictor of ciprofloxacin nonsusceptibility (adjusted OR, 14.4; 95% CI, 8.60–22.90; *p* < 0.001). *E. coli* showed a higher odds ratio than non-*E. coli* (adjusted OR, 6.0; 95% CI, 1.70–12.60; *p* = 0.001). Early-onset showed a non-significant association (adjusted OR, 0.64; 95% CI, 0.39–1.05; *p* = 0.076), and male sex was not associated with resistance (adjusted OR, 1.11; 95% CI, 0.65–1.91; *p* = 0.697). Compared with the during-COVID-19 period, the pre-COVID-19 period was associated with significantly lower odds of ciprofloxacin nonsusceptibility (OR, 0.52; 95% CI, 0.31–0.89; *p* = 0.016), whereas the post-COVID-19 period did not differ significantly (OR, 1.40; 95% CI, 0.80–2.47; *p* = 0.24). Adjusted odds ratios and confidence intervals are summarized in Figure 3 (log-scale) and Supplementary Table S5.

## 3. Discussion

The global surge in antibiotic resistance, driven by overuse, poses a significant threat to healthcare. Eastern Europe and Asia report higher resistance rates than Western Europe [14], and Korea shows similarly elevated levels, underscoring the importance of reducing unnecessary antimicrobial exposure and improving stewardship in pediatric care. In this study, the resistance rates to first-line empirical treatments for pediatric UTIs—ampicillin, TMP/SMX, and amoxicillin/clavulanate—were 65.2%, 33.5%, and 11.2%, respectively, consistent with national multicenter data [15,16]. Carbapenem resistance remained 0% throughout the study period. Nationally, the pediatric carbapenem resistance rate is <10% [15], contrasting with the rates of 6.4–8.6% and >20% in overseas reports of pediatric UTI and in bacteremia [17], respectively. Prior multicenter studies reported *E.coli* carbapenem resistance at 0.3–1.2% (low) versus *K. pneumoniae* resistance at 0.4–16.8% (high) [18]. Of note, 89.6% of the pathogens in this study being *E. coli* and enrollment being limited to first-episode UTI patients, excluding carbapenem resistance risk factors (antibiotic use within 2 weeks, urinary catheter insertion, or hospitalization), likely contributed to the low resistance [19]. Nonetheless, further analyses, including strain genotyping, are required to elucidate institution-specific features. Although resistance appeared to increase over time, such trends must be interpreted cautiously because temporal fluctuations may reflect environmental confounders (e.g., influenza seasons and pandemic-related healthcare disruptions) as well as inherent limitations of single-center surveillance. These contextual factors highlight the need to interpret resistance trends within a broader ecological framework rather than attributing them solely to antibiotic consumption patterns.

In our longitudinal evaluation across pre-, during-, and post-pandemic periods, AMR remained persistently high despite a marked reduction in pediatric outpatient antibiotic prescribing. Overall, high resistance rates were observed not only for ciprofloxacin but also for ampicillin, TMP/SMX, and amoxicillin/clavulanate, which are commonly used as first-line empirical therapies in pediatric UTIs. In Korea, a multicenter study reported that third-generation cephalosporin resistance among pediatric UTI pathogens rose significantly from about 10% in the early 2010s to over 30% after 2020 [15]. In Lebanon, a decade-long surveillance study of hospitalized children with urinary tract infections showed a rising trend of multidrug-resistant uropathogens, particularly against cephalosporins and fluoroquinolones, increasing again after 2019 and reaching 67.9% in 2021 (*p* = 0.248) [20]. Similarly, in Japan, despite a nearly 50% reduction in pediatric oral antibiotic use between 2016 and 2020 [21,22], resistance to β-lactams and TMP/SMX remained persistently high, supporting the notion that reduced antibiotic consumption alone may not lead to an immediate decrease in resistance [23]. The persistent high resistance rate observed across regions likely reflects broader community-level selective pressures and transmission dynamics that extend beyond pediatric prescribing. These findings support the possibility that AMR trends may be driven by broader ecological and transmission dynamics rather than pediatric prescribing alone.

One of the most notable findings in our study was the significant increase in ciprofloxacin resistance, despite ciprofloxacin being rarely used in pediatric patients. Ciprofloxacin susceptibility declined significantly during the COVID-19 period (*p* = 0.003). ITS analysis demonstrated an immediate 12.1-percentage-point rise in ciprofloxacin nonsusceptibility at the onset of the pandemic (2020 Q1), with stabilization, thereafter, indicating a step-change rather than a gradual trend. This abrupt shift suggests an ecological influence temporally aligned with the pandemic period rather than a pattern attributable to pediatric prescribing. We selected ciprofloxacin for ITS modeling because it exhibited the most robust and statistically significant temporal change and is recognized as a marker of MDR in Gram-negative urinary pathogens. The clear step-change observed supports the hypothesis that external pressures—such as adult prescribing patterns and community reservoirs—may contribute to pediatric resistance patterns.

During the COVID-19 pandemic, pediatric outpatient antibiotic prescriptions declined markedly [7,8], while adult inpatient use of fluoroquinolones and broad-spectrum agents increased, potentially affecting community AMR ecology [24,25]. This contrasting pattern supports the possibility that pediatric resistance trends may have been influenced by broader community-level antimicrobial pressures rather than pediatric prescribing alone. A particularly informative finding was the pronounced decline in ciprofloxacin susceptibility among infants younger than 100 days, decreasing from 80.3% to 66.4% (*p* = 0.026). Age-specific differences further support this interpretation. The pronounced increase in ciprofloxacin resistance among infants younger than 100 days is particularly meaningful, as this group has minimal direct antibiotic exposure. Therefore, the resistance pattern observed in this cohort is consistent with the possibility of perinatal or early household transmission, which corresponds to previous reports showing that maternal-to-neonate transmission exerts its strongest impact during the first weeks of life and remains relevant up to 2–3 months of age [26,27,28]. In contrast, the 100-day–24-month age group was more heterogeneous, with varied clinical backgrounds and patterns of antibiotic exposure. Such diversity may have led to a wider distribution of nonsusceptibility, thereby attenuating the statistical significance compared with the more uniform and high-risk cohort of infants under 100 days of age. Therefore, the marked resistance in this group likely reflects the broader burden of resistance circulating in the community.

In our multivariable analysis, ESBL production emerged as an independent predictor of ciprofloxacin resistance, even after adjusting for age, sex, and pathogen species. Moreover, the significant association with *E. coli* underscores its predominance as a high-risk pathogen in children. This finding is consistent with evidence that ciprofloxacin resistance in *E. coli* is strongly associated with the dissemination of high-risk clonal lineages, particularly ST131 and ST1193, which frequently carry CTX-M-type ESBLs [29,30,31]. In Korea, although pediatric data are limited, molecular studies have detected ST131 among ESBL-producing *E. coli* in children, and Yun et al. reported that ST131 accounted for 31–36% of the lineages [32]. Among community-acquired UTIs in adults, ciprofloxacin-resistant *E. coli* isolates accounted for 28.7% of ST131 and 13.1% ST1193 lineages [33]. These epidemiologic findings are consistent with molecular reports describing the frequent co-occurrence of fluoroquinolone resistance determinants (e.g., gyrA, parC mutations, plasmid-mediated qnr genes, aac(6′)-Ib-cr) with ESBL-encoding plasmids such as CTX-M [34,35]. These co-resistance mechanisms may partly explain the extensive multidrug resistance observed in our isolates, and ciprofloxacin nonsusceptibility may serve as a sentinel marker of multidrug resistance.

The persistence of resistance, especially to ciprofloxacin, TMP/SMX, and third-generation cephalosporins, highlights the shrinking therapeutic window for pediatric UTI. Although fluoroquinolones are generally avoided in children, resistance remains epidemiologically important because it reflects the community dissemination of high-risk lineages that frequently harbor ESBLs. From a public health perspective, despite reduced pediatric prescribing, the persistence of ciprofloxacin resistance suggests that AMR may be influenced not only by prescribing patterns but also by broader ecological and transmission dynamics.

This study has several limitations. First, it was conducted at a single tertiary care center, which may limit the generalizability of our findings to other settings or community-acquired infections. Second, we did not perform molecular typing of the isolates, and this precluded direct confirmation of the involvement of high-risk clonal lineages such as *E. coli* ST131 or plasmid-mediated resistance determinants. Third, the retrospective design and restriction to hospitalized febrile UTI cases may not fully reflect the resistance patterns in outpatients or milder infections. Despite these limitations, our findings are consistent with national and international surveillance data, supporting the validity of the observed resistance trends. Future multicenter studies incorporating molecular typing, including both inpatient and outpatient populations, are needed to clarify the pathways and ecological pressures underlying fluoroquinolone resistance in children.

## 4. Materials and Methods

### 4.1. Study Design and Population

This study was conducted at Pusan National University Yangsan Hospital (PNUYH), the only designated children’s hospital serving the Busan, Ulsan, and Gyeongsangnam-do regions and functioning as the primary tertiary referral center for pediatric care. The hospital receives approximately 50,000 pediatric admissions annually. Between 24 November 2008, and 31 August 2023, a total of 2349 children under 24 months of age were hospitalized with febrile UTI. This broad regional catchment supports the representativeness of our study cohort and reflects the epidemiology of pediatric UTIs in the area.

This retrospective study reviewed the electronic medical records of patients diagnosed with febrile UTI at Pusan National University Yangsan Hospital (PNUYH), a tertiary center, from 24 November 2008 to 31 August 2023. The included patients were under 24 months of age at diagnosis, had their first UTI, and were hospitalized at PNUYH. The exclusion criteria were as follows: (1) hospital-acquired UTI, (2) UTI associated with indwelling urinary catheter placement, (3) history of congenital anomalies of the kidney and urinary tract (CAKUT), (4) concurrent or underlying diseases, and (5) insufficient or unclear data.

### 4.2. Definition and Data Collection

UTI was defined as the presence of fever (≥38.0 °C) accompanied by pyuria or bacteriuria on urinalysis. Pyuria was characterized as urine white blood cell (WBC) count ≥5 per high-power field (HPF) or positive result for leukocyte esterase, nitrite, or bacteriuria on urine dipstick testing. Definitive diagnosis was confirmed by urine culture demonstrating growth of a single pathogen at ≥50,000 colony-forming units (CFU)/mL. All urine specimens were collected via sterile catheterization.

The baseline patient characteristics included sex, age at onset, urine culture results, and temporal changes in antibiotic resistance patterns. Comparative analyses were conducted by dividing the patients into two primary groups based on age at onset: the early-onset group was defined as patients with the first UTI occurring within the first 100 days of life, and the delayed-onset group was defined as patients with the first UTI occurring between 100 days and 24 months of age. Subgroup analyses were conducted by stratifying patients according to the onset period (2018–2019, pre-COVID-19 period; 2020–2021, COVID-19 period; 2022–2023, post-COVID-19 period) to assess the potential impact of the COVID-19 pandemic on pediatric UTI epidemiology and antibiotic resistance patterns. These periods were defined according to the national COVID-19 response phases of the Korea Disease Control and Prevention Agency (KDCA), which reported the first domestic case in January 2020 and downgraded COVID-19 from a Class 1 to Class 2 infectious disease in April 2022. This division accounted for changes in healthcare delivery, antibiotic prescription practices, and infection control measures during the pandemic as well as temporal trends in antibiotic resistance.

### 4.3. Microbiological Methods

Urinary cultures and urinary pathogen identification were performed using the VITEK^®^ 2 automated system (bioMérieux, Hazelwood, MO, USA). Antibiotic susceptibility testing and ESBL identification were conducted using antibiotic susceptibility test cards with the VITEK^®^ 2 system, following Clinical and Laboratory Standards Institute guidelines. Nonsusceptibility was defined as not susceptible (Intermediate + Resistant; I + R). The resistance profiles to the following antibiotics were evaluated: amoxicillin/clavulanate, ampicillin, amikacin, aztreonam, ceftazidime, cefazolin, ciprofloxacin, cefotaxime, ertapenem, cefepime, gentamicin, imipenem, TMP/SMX, tigecycline, and piperacillin/tazobactam. ESBL positivity was also assessed.

### 4.4. Statistical Analysis

Data were presented as numbers (percentages) for categorical variables and as means with standard deviations (SD) for continuous variables. Continuous data were compared using Student’s *t*-tests, while categorical data were analyzed using the χ^2^ test. A linear-by-linear association method was employed to compare the ordinal scales of three or more independent variables. Temporal changes in antimicrobial resistance were assessed using ITS analysis with segmented regression, estimating levels and slope changes across the pre-, during-, and post-pandemic periods. Multivariate logistic regression was performed to identify the independent predictors of ciprofloxacin susceptibility. All statistical analyses were conducted using SPSS (version 28.0; SPSS Inc., Chicago, IL, USA) and R version 4.3.1 (R Foundation for Statistical Computing, Vienna, Austria) with the itsa package. The Python software (version 3; Python Software Foundation, Wilmington, DE, USA) was used to visualize the data. A *p* value < 0.05 was considered statistically significant.

## 5. Conclusions

Despite a marked decline in pediatric antibiotic prescribing during the COVID-19 pandemic, antimicrobial resistance in urinary pathogens among children under 24 months remained persistently high, with ciprofloxacin resistance increasing. High resistance to ampicillin, TMP/SMX, amoxicillin/clavulanate, and ciprofloxacin underscores the narrowing therapeutic options for pediatric UTIs. These findings indicate that reductions in pediatric antibiotic use alone are insufficient to reverse resistance trends, as community-level antimicrobial pressures and cross-age transmission dynamics likely contribute to their persistence. Integrated stewardship across adult and pediatric populations, together with genomic surveillance of high-risk clones such as *E. coli* ST131, will be essential to curb further dissemination.

## Figures and Tables

**Table 1:** Comparison of clinical characteristics between early-onset and delayed-onset groups with febrile urinary tract infection (UTI).

	Early-Onset (*n* = 605)	Delayed-Onset (*n* = 544)
Male sex	514 (85.0)	335 (61.6)
Fever duration (days)	1.8 ± 1.0	2.9 ± 1.6
Initial Laboratory findings		
WBC (×10^3^/μL)	14.6 ± 5.5	16.9 ± 6.1
Hemoglobin (g/dL)	10.3 ± 1.2	11.2 ± 0.8
Platelet (×10^3^/μL)	437.0 ± 120.5	396.0 ± 109.6
ESR (mm/h)	19.3 ± 17.8	27.5 ± 24.0
CRP (mg/dL)	4.1 ± 3.7	5.3 ± 4.7
Urinary structural anomalies in USG	292/433 (67.4)	218/364 (59.9)
Cortical defect in DMSA scan	117/378 (31.0)	183/417 (43.9)

Values are presented as number (%) or mean ± S.D (standard deviation); UTI, urinary tract infection; WBC, white blood cell; ESR, erythrocyte sedimentation rate; CRP, C-reactive protein; USG, ultrasonography; DMSA, dimercaptosuccinic acid.

**Table 2:** Comparison of antibiotic sensitivity rate (%) between early-onset and delayed-onset group.

	Early-Onset (*n* = 605)	Delayed-Onset (*n* = 544)	*p* Value *
Amoxicillin/Clavulanate	430/573 (75.0)	366/512 (71.5)	0.186
Ampicillin	207/573 (36.1)	166/506 (32.8)	0.253
Amikacin	572/573 (99.8)	514/516 (99.6)	0.503
Aztreonam	470/573 (82.0)	414/516 (80.2)	0.450
Ceftazidime	465/573 (81.2)	411/515 (79.8)	0.576
Cefazolin	367/520 (70.6)	325/468 (69.4)	0.698
Ciprofloxacin	415/542 (76.6)	388/474 (81.9)	0.039
Cefotaxime	457/575 (79.5)	407/517 (78.7)	0.759
Ertapenem (*n* = 969)	508/508 (100)	461/461 (100)	-
Cefepime (*n* = 1087)	469/571 (82.1)	418/516 (81.0)	0.631
Gentamycin (*n* = 1086)	445/571 (77.9)	415/515 (80.6)	0.283
Imipenem (*n* = 1083)	569/569 (100)	514/514 (100)	-
TMP/SMX (*n* = 1091)	383/576 (66.5)	343/515 (66.6)	0.970
Tigecycline (*n* = 989)	521/521 (100)	464/468 (99.1)	0.134
PIP/TAZ (*n* = 1027)	519/540 (96.1)	457/487 (93.8)	0.094
ESBL (*n* = 1036)	448/551 (81.3)	388/485 (80)	0.595

Values are presented as number (%); * is χ^2^ test.

**Table 3:** Changes in antibiotic susceptibility in pre-, during-, and post-COVID-19 period.

	Pre-COVID-19 (*n* = 274)	During-COVID-19 (*n* = 171)	Post-COVID-19 (*n* = 158)	*p* Value *
Amoxicillin/clavulanate	189/269 (70.3)	112/166 (67.5)	85/145 (58.6)	0.021
ampicillin	79/267 (29.6)	53/162 (32.7)	48/144 (33.3)	0.402
Amikacin	269/269 (100)	165/166 (99.4)	146/146 (100)	0.796
Aztreonam	214/269 (79.6)	123/166 (74.1)	91/146 (62.3)	<0.001
Ceftazidime	212/269 (78.8)	121/166 (72.9)	117/146 (80.1)	0.974
Cefazolin	188/269 (69.9)	107/166 (64.5)	100/146 (68.5)	0.635
Ciprofloxacin	223/274 (81.4)	116/170 (68.2)	110/158 (69.6)	0.003
Cefotaxime	206/269 (76.6)	120/167 (71.9)	116/146 (79.5)	0.686
Ertapenem	269/269 (100)	166/166 (100)	146/146 (100)	-
Cefepime	216/269 (80.3)	123/166 (74.1)	118/146 (80.8)	0.877
Gentamicin	198/269 (73.6)	130/166 (78.3)	116/147 (78.9)	0.188
Imipenem	269/269 (100)	165/165 (100)	141/141 (100)	-
TMP/SMX	165/269 (61.3)	104/167 (62.3)	99/147 (67.3)	0.249
Tigecycline	273/273 (100)	171/171 (100)	156/157 (99.4)	0.147
PIP/TAZ	254/269 (94.4)	160/166 (96.4)	140/145 (96.6)	0.276
ESBL negative	208/261 (79.7)	114/157 (72.6)	104/132 (78.8)	

Values are presented as number (%); * is χ^2^ test. COVID-19 virus disease 2019; TMP/SMX, trimethoprim and sulfamethoxazole; PIP/TAZ, piperacillin and tazobactam; ESBL, Extended-spectrum β-lactamase.

## Data Availability

The original contributions presented in this study are included in the article. Further inquiries can be directed to the corresponding author.

## Supplementary Materials

The following supporting information can be downloaded at: https://www.mdpi.com/article/10.3390/antibiotics14121243/s1, Table S1: Baseline characteristics of first attack febrile; Table S2: Pathogen of first attack febrile UTI; Table S3: Comparison of UTI pathogen between the early onset and delayed onset; Table S4: Interrupted Time-Series Analysis of Ciprofloxacin Nonsusceptibility; Table S5: Multivariable logistic regression for predictors of ciprofloxacin resistance.

## Abbreviations

The following abbreviations are used in this manuscript:

AMR	Antimicrobial resistance
ALT	Alanine aminotransferase
AST	Aspartate Transaminase
BUN	Blood Urea Nitrogen
CFU/mL	Colony-forming units per milliliter
CI	Confidence interval
CLSI	Clinical and Laboratory Standards Institute
COVID-19	Coronavirus disease 2019
CRP	C-reactive protein
DMSA	Dimercaptosuccinic Acid
*E. coli*	*Escherichia coli*
ESBL	Extended-spectrum β-lactamase
ESR	Erythrocyte Sedimentation Rate
HPF	High power field
ITS	Interrupted time-series
OR	Odds ratio
PIP/TAZ	Piperacillin and tazobactam
SD	Standard deviation
TMP/SMX	Trimethoprim/sulfamethoxazole
USG	Ultrasonography
UTI	Urinary tract infection
WBC	White blood cell

## References

[1] Shaikh N., Morone N.E., Bost J.E., Farrell M.H. (2008). Prevalence of urinary tract infection in childhood: A meta-analysis. Pediatr. Infect. Dis. J..

[2] Bryce A., Hay A.D., Lane I.F., Thornton H.V., Wootton M., Costelloe C. (2016). Global prevalence of antibiotic resistance in paediatric urinary tract infections caused by *Escherichia coli* and association with routine use of antibiotics in primary care: Systematic review and meta-analysis. BMJ.

[3] Tamma P.D., Rodriguez-Bano J. (2017). The Use of Noncarbapenem β-Lactams for the Treatment of Extended-Spectrum β-Lactamase Infections. Clin. Infect. Dis..

[4] Langford B.J., So M., Raybardhan S., Leung V., Soucy J.R., Westwood D., Daneman N., MacFadden D.R. (2021). Antibiotic prescribing in patients with COVID-19: Rapid review and meta-analysis. Clin. Microbiol. Infect..

[5] World Health Organization (2024). WHO Reports Widespread Overuse of Antibiotics in Patients Hospitalized with COVID-19. https://www.who.int/news/item/26-04-2024-who-reports-widespread-overuse-of-antibiotics-in-patients--hospitalized-with-covid-19.

[6] Ryu S., Hwang Y., Ali S.T., Kim D.S., Klein E.Y., Lau E.H.Y., Cowling B.J. (2021). Decrease in broad-spectrum antibiotic prescriptions in children during the COVID-19 pandemic in Korea. J. Infect. Dis..

[7] Jeong Y.-I., Lee H.-Y., Lee S., Jeong G.Y., Kim S.H., Kim S., Seo S.-H., Shin N.-R. (2025). Korea’s National Action Plan on Antimicrobial Resistance: Focusing on the Appropriate Use of Antibiotics. Infect. Chemother..

[8] Park J., Kang H.M. (2024). National level cross-sectional study on antibiotic use in children during the pre- and early COVID-19 eras. Antibiotics.

[9] Hong J.H., Paek S.H., Kim T., Kim S., Ko E., Ro Y.S., Kim J., Kwon J.H. (2023). Characteristics of pediatric emergency department visits before and during the COVID-19 pandemic: A report from the National Emergency Department Information System (NEDIS) of Korea, 2018–2022. Clin. Exp. Emerg. Med..

[10] Woo B., Jung Y., Kim H.S. (2019). Antibiotic Sensitivity Patterns in Children with Urinary Tract Infection: Retrospective Study over 8 Years in a Single Center. Child. Kidney Dis..

[11] Yong H.T., Park S.C., Lee J.W. (2017). Changes in Causative Organisms and Antimicrobial Susceptibility of the Urinary Tract Infection. J. Korea Acad.-Ind. Coop. Soc..

[12] Logan L.K., Weinstein R.A. (2017). The Epidemiology of Carbapenem-Resistant Enterobacteriaceae: The Impact and Evolution of a Global Menace. J. Infect. Dis..

[13] Banerjee R., Robicsek A., Kuskowski M.A., Porter S., Johnston B.D., Sokurenko E., Tchesnokova V., Price L.B., Johnson J.R. (2013). Molecular epidemiology of Escherichia coli sequence type 131 and Its H30 and H30-Rx subclones among extended-spectrum-β-lactamase-positive and -negative E. coli clinical isolates from the Chicago Region, 2007 to 2010. Antimicrob. Agents Chemother..

[14] Romandini A., Pani A., Schenardi P.A., Pattarino G.A.C., De Giacomo C., Scaglione F. (2021). Antibiotic Resistance in Pediatric Infections: Global Emerging Threats, Predicting the Near Future. Antibiotics.

[15] Park P.G., Lim S.H., Song J.Y., Ahn Y.H., Kim S.H., Kang H.G. (2025). Trends in antibiotic resistance of urinary tract infections in young children, 2010–2023. Pediatr. Neonatol..

[16] Kim J.-A., Song S.A., Kim S., Park S., Woo K., Kim Y.K. (2025). A multicenter study on antimicrobial resistance in bloodstream pathogens isolated in Korea: A survey study. Ann. Clin. Microbiol. Antimicrob..

[17] Begum R.S. (2024). Global Perspectives on Pediatric Antimicrobial Resistance: A Systematic Literature Review. Medtigo J. Med..

[18] Jo S.B., Ahn S.T., Joo H.J., Kim J.W., Oh M.M. (2025). Carbapenem Resistance and ESBL-Producing Enterobacteriaceae in Patients with Urological Infections from 2012 to 2021 in Three Korean Hospitals. Diagnostics.

[19] Bandac C.A., Ristescu C., Onofrei P., Miftode I.L., Radu R., Boiculese V.L., Pauna A.R., Pantilimonescu T.F., Luduşanu A., Radu V.D. (2025). Assessment of Factors Contributing to Multidrug Resistance in Urinary Tract Infections: Focus on Carbapenem Resistance. Antibiotics.

[20] El Zein Z., Boutros C.F., El Masri M., El Tawil E., Sraj M., Salameh Y., Ghadban S., Salameh R., El Baasiri S., Haddara A. (2025). The challenge of multidrug resistance in hospitalized pediatric patients with urinary tract infections. Front. Cell Infect. Microbiol..

[21] Otake S., Shoji T., Yamada K., Kimura M., Myojin S., Kamiyoshi N., Ochi F., Nezu M., Ishida A., Miyairi I. (2024). Trend in antibiotic prescription at pediatric primary emergency medical centers in Japan: A multi-center, cross-sectional study. J. Infect. Chemother..

[22] Muramatsu D., Yanai T., Yoshida S., Kawakami K. (2024). Prescribing Pattern and Efficacy of Oral Antibiotics for Pediatric Urinary Tract Infections in Japan: A Descriptive Study Using a Nationwide Claims Database. Pediatr. Infect. Dis. J..

[23] Ono A., Koizumi R., Tsuzuki S., Asai Y., Ishikane M., Kusama Y., Ohmagari N. (2022). Antimicrobial Use Fell Substantially in Japan in 2020-The COVID-19 Pandemic May Have Played a Role. Int. J. Infect. Dis..

[24] Jeon K., Jeong S., Lee N., Park M.-J., Song W., Kim H.-S., Kim H.S., Kim J.-S. (2022). Impact of COVID-19 on Antimicrobial Consumption and Spread of Multidrug-Resistance in Bacterial Infections. Antibiotics.

[25] Choi Y., Kang M., Shin D.H., Jung J., Choi S.J., Kim N.-H., Moon S.M., Song K.-H., Kim E.S., Jung J. (2023). Antibiotic Prescription in Patients With Coronavirus Disease 2019: Analysis of National Health Insurance System Data in the Republic of Korea. J. Korean Med. Sci..

[26] Chan G.J., Lee A.C., Baqui A.H., Tan J., Black R.E. (2013). Risk of early-onset neonatal infection with maternal infection or colonization: A global systematic review and meta-analysis. PLoS Med..

[27] Denkel L.A., Schwab F., Kola A., Leistner R., Garten L., von Weizsäcker K., Geffers C., Gastmeier P., Piening B. (2014). The mother as most important risk factor for colonization of very low birth weight (VLBW) infants with extended-spectrum β-lactamase-producing Enterobacteriaceae (ESBL-E). J. Antimicrob. Chemother..

[28] Rettedal S., Löhr I., Bernhoff E., Natås O., Sundsfjord A., Øymar K. (2015). Extended-spectrum β-lactamase-producing Enterobacteriaceae among pregnant women in Norway: Prevalence and maternal–neonatal transmission. J. Perinatol..

[29] Johnson J.R., Johnston B., Clabots C., Kuskowski M.A., Castanheira M. (2010). Escherichia coli sequence type ST131 as the major cause of serious multidrug-resistant E. coli infections in the United States. Clin. Infect. Dis..

[30] Nicolas-Chanoine M.H., Bertrand X., Madec J.Y. (2014). Escherichia coli ST131, an intriguing clonal group. Clin. Microbiol. Rev..

[31] Tchesnokova V., Radey M., Chattopadhyay S., Larson L., Weaver J.L., Kisiela D., Sokurenko E.V. (2019). Pandemic fluoroquinolone resistant Escherichia coli clone ST1193 emerged via simultaneous homologous recombinations in 11 gene loci. Proc. Natl. Acad. Sci. USA.

[32] Yun K.W., Lee M.K., Kim W., Lim I.S. (2017). Uropathogenic Escherichia coli ST131 in urinary tract infections in children. Korean J. Pediatr..

[33] Kim B., Seo M.-R., Kim J., Kim Y., Wie S.-H., Ki M., Cho Y.K., Lim S., Lee J.S., Kwon K.T. (2020). Molecular Epidemiology of Ciprofloxacin-Resistant Escherichia coli Isolated from Community-Acquired Urinary Tract Infections in Korea. Infect. Chemother..

[34] Robicsek A., Jacoby G.A., Hooper D.C. (2006). The worldwide emergence of plasmid-mediated quinolone resistance. Lancet Infect. Dis..

[35] Rodríguez-Martínez J.M., Cano M.E., Velasco C., Martínez-Martínez L., Pascual A. (2011). Plasmid-mediated quinolone resistance: An update. J. Infect. Chemother..

