# Supplementary material for: Changes in Antimicrobial Resistance in Pediatric Urinary Pathogens Before, During, and After the COVID-19 Pandemic
Source: Antibiotics (Basel). 2025 Dec 9;14(12):1243. doi: 10.3390/antibiotics14121243 (PMC12729262; doi:10.3390/antibiotics14121243)
Supplement: Supplementary file 1 [file antibiotics-14-01243-s001.zip › antibiotics-3971529-supplementary.pdf]

Table S1. Baseline characteristics of first attack febrile UTI

Contents	Results (N =1,149)
Male: Female	849:300
Mean age at diagnosis (days)	121.1±91
Number of diagnostic age <100days	605 (52.7%)
Number of 100 days≤ diagnostic age <24monts	544 (47.3%)
Duration of admission (days)	5.4±2.4
Total fever duration (days)	2.3±1.4
Pre-admission (days)	1.9±1.2
Post admission (days)	0.4±0.7
Initial Lab findings	
WBC ($x10^3/\mu L$)	15.70±5.90
Hemoglobin (g/dL)	10.7±1.2
Platelet ($x10^3/\mu L$)	417.6±117.2
ESR (mm/hr)	23.3±21.4
CRP (mg/dL)	4.7±4.2
BUN (mg/dL)	9.0±3.2
Creatinine (mg/dL)	0.26±0.08

AST (IU/L)	47.8±67.1
ALT (IU/L)	38.7±71.0
Urinary structural anomalies in USG (n=797)	510 (64.0%)
Cortical defect in DMSA (n=795)	300 (37.7%)
Number of *E*. coli UTI	1,029 (89.6%)
Number of ESBL (+) pathogen	200 (19.3%)

UTI, Urinary tract infection; WBC, White blood cell; ESR, Erythrocyte Sedimentation Rate; CRP, C-reactive protein; BUN, Blood Urea Nitrogen; AST, Aspartate Transaminase; ALT, Alanine aminotransferase; USG, Ultrasonography; DMSA, Dimercaptosuccinic Acid; *E*. coli, *Escherichia coli*; ESBL, Extended-spectrum β-lactamase

Table S2. Pathogen of first attack febrile UTI

**Antigen**	**Cases (N, %)**
Citrobacter freundii	2 (0.17)
Citrobacter koseri	7 (0.60)
Escherichia coli	1029 (89.6)
Enterobacter cloacae	3 (0.26)
Enterococcus faecalis	22 (1.9)
Enterococcus faecium	5 (0.44)
Enterococcus	2 (0.17)
Klebsiella aerogenes	32 (2.8)
Klebsiella oxytoca	7 (0.61)
Klebsiella pneumoniae	30 (2.6)
Morganella morganii	1 (0.09)
Proteus mirabilis	3 (0.26)

UTI, Urinary tract infection

Table S3. Comparison of UTI pathogen between the early onset and delayed onset

	Early onset (N=605)	Delayed onset (N=544)	*p* value
Citrobacter spp.	5 (0.82%)	4 (0.74%)	0.861
Citrobacter freundii	1 (0.2%)	1 (0.2%)	0.940
Citrobacter koseri	4 (0.7%)	3 (0.6%)	0.811
Escherichia coli	542 (89.6%)	487 (89.5%)	0.971
Enterobacter cloacae	1 (0.2%)	2 (0.4%)	0.502
Enterococcus spp.	20 (3.3%)	9 (1.7%)	0.075
Enterococcus faecalis	16 (2.6%)	6 (1.1%)	0.057
Enterococcus faecium	3 (0.5%)	2 (0.4%)	0.742
Enterococcus	1 (0.2%)	1 (0.2%)	0.940
Klebsiella spp.	34 (5.6%)	35 (6.4%)	0.562
Klebsiella aerogenes	16 (2.6%)	16 (2.9%)	0.760
Klebsiella oxytoca	2 (0.3%)	5 (0.9%)	0.201
Klebsiella pneumoniae	16 (2.6%)	14 (2.6%)	0.940
Morganella morganii	0 (0)	1 (0.2%)	0.291
Proteus mirabilis	0 (0)	3 (0.6%)	0.067

UTI, Urinary tract infection

Table S4. Interrupted Time-Series Analysis of Ciprofloxacin Nonsusceptibility

Segment	Estimate (%)	95% CI	p-value
Pre-pandemic slope	+0.048	−0.022 to +0.118	0.179
Immediate level change at 2020-Q1	+0.603	+0.097 to +1.109	0.019
Slope change during pandemic	−0.081	−0.187 to +0.025	0.135
Immediate level change at 2022-Q1	+0.133	−0.698 to +0.964	0.754
Slope change post-pandemic	+0.013	−0.152 to +0.179	0.873

Q1, quarter 1

Table S5. Multivariable logistic regression for predictors of ciprofloxacin resistance

Variable	OR	95% CI	p-value
Age < 100days vs. 100d<24months	0.64	0.39–1.05	0.076
Female vs. Male	1.11	0.65–1.91	0.697
Post-COVID-19 vs. During	1.40	0.80–2.47	0.24
Pre-COVIVD-19 vs. During	0.52	0.31–0.89	0.016
E. coli vs. Non-*E.coli*	6.00	1.70–12.60	0.001
ESBL positive	14.40	8.60–22.90	<0.001

COVID-19, coronavirus disease 2019; *E. coli*, *Escherichia coli*; ESBL, Extended-spectrum β-lactamase
